# The Microbiome of the ‘Williams’ Pear Variety Grown in the Organic Orchard and Antifungal Activity by the Autochthonous Bacterial and Yeast Isolates

**DOI:** 10.3390/microorganisms10071282

**Published:** 2022-06-24

**Authors:** Tamara Janakiev, Tanja Berić, Tatjana Stević, Slaviša Stanković, Jasmina Bačić, Helena Majstorović, Djordje Fira, Ivica Dimkić

**Affiliations:** 1 Faculty of Biology, University of Belgrade, Studentski trg 16, 11158 Belgrade, Serbia; tamara.janakiev@bio.bg.ac.rs (T.J.); tanjab@bio.bg.ac.rs (T.B.); slavisas@bio.bg.ac.rs (S.S.); fira@bio.bg.ac.rs (D.F.); 2 Institute for Medicinal Plants Research “Dr. Josif Pančić”, Tadeuša Košćuška 1, 11000 Belgrade, Serbia; tanjasomosa@gmail.com; 3 PSS “Tamiš” Institute, Agriculture Extension Service Province of Vojvodina, Novoseljanski put 33, 26000 Pančevo, Serbia; jasminabacic4691@gmail.com (J.B.); majstorovic@institut-tamis.rs (H.M.)

**Keywords:** microbiome, Williams’s pear variety, antifungal activity, biological control

## Abstract

The total diversity of bacterial and fungal communities associated with the phyllosphere (fruits and leaves) of the ‘Williams’ pear variety was analyzed in two phenological stages during fruit development and maturation. The antagonistic potential of autochthonous bacterial and yeast isolates against phytopathogenic fungi was also evaluated. A metabarcoding approach revealed *Pantoea*, *Sphingomonas*, *Hymenobacter*, *Massilia*, and *Pseudomonas* as dominant bacterial constituents of the pear phyllosphere, whilst most abundant among the fungal representatives identified were *Metschnikowia*, *Filobasidium*, *Aureobasidium* *pullulans*, *Botrytis cinerea*, and *Taphrina*. The traditional culturable approach revealed that the *Pseudomonas* genus with *P. graminis*, *P. putida*, and *P. congelans* was most prevalent. The most frequently cultivated fungal representatives belonged to the genus *Fusarium* with six identified species. A broad range of the antagonistic activity was detected for the *Hannaella luteola* and *Metschnikowia pulcherrima* yeasts, significantly affecting the growth of many fungal isolates in the range of 53–70%. *Fusarium sporotrichioides* was the most susceptible fungal isolate. The autochthonous antagonistic yeasts *H. luteola* and *M. pulcherrima* might be powerful biological control agents of postharvest diseases caused by *Fusarium* spp. and common pathogens like *Monilinia laxa*, *Botrytis cinerea*, *Alternaria tenuissima*, and *Cladosporium cladosporioides*.

## 1. Introduction

*Pyrus communis* (L.), a European pear, is one of the most common species of the pome fruit group cultivated worldwide [1]. Among the most widespread pear varieties in Europe is ‘Williams’, also known as the ‘Bartlett’ pear in the USA, with an annual production of between 250,000 and 300,000 ha in the last few years [2]. Due to its pleasant aroma, it is much appreciated as raw material for the production of high-quality Williams brandy [3]. In general, the quality of the fruit and brandy that it produces depends on several factors including microorganisms [4]. Current knowledge about microbiota of the ’Williams’ variety is mainly focused on bacterial and fungal species as causative agents of plant diseases with further negative effects on pear fruit yield and economical losses [5,6].

Studies of total microbial diversity associated with the ‘Williams’ variety, and pears generally, were discussed in several studies that mainly focused on rhizosphere soil, twigs, and postharvest microbiota. Total bacterial diversity was analyzed by a next-generation sequencing approach in the floral nectar of the ‘Conference’ variety [7]. NGS was used to assess the bacterial diversity of rhizosphere soil of the ‘Krystalli’ Greek pear variety [8]. Bacterial populations from surface soil of ‘Blanquilla’, ‘Conference’, and ‘Williams’ varieties were characterized by PCR-denaturing gradient gel electrophoresis [9]. Furthermore, the cultivable approach was used to assess the composition of fungal communities residing on twigs of the ‘Williams’ variety [10], and fungi associated with postharvest decay on the fruit of ‘Krystalli’ variety which was characterized by NGS [11]. To the best of our knowledge, a comprehensive study on the fungal and bacterial communities of the ‘Williams’ variety phyllosphere (fruit and leaves) analyzed with the NGS approach has not been published.

Phyllosphere microorganisms play an important role in plant health, such as nutrient acquisition, plant growth hormone production, abiotic stress tolerance, and biocontrol abilities of the plant pathogens. Biocontrol as a strategy for suppression of phytopathogens by beneficial phyllosphere microorganisms is based on many mechanisms, such as competition, antibiosis, induction of host immune response, etc. [12,13]. Using biocontrol as an environmentally friendly alternative approach for the treatment of phytopathogens is becoming a public demand in order to produce healthier food without the application of chemical pesticides [14]. Phytopathogenic fungi reduce up to 80% of crop yield, as well as quality [15]. The most important pathogens of pears are fungi belonging to *Fusarium* and *Monilinia* genera [16]. Representatives of the *Fusarium* genus are causal agents of the most significant crop diseases worldwide causing up to 50% yield loss. They synthesize toxic secondary metabolites, known as mycotoxins, which are compounds that are easily accumulated in the tissues of cereals and fruits and become life-threatening or severely impair human and animal biological systems [17]. Furthermore, brown rot caused by *Monilinia* spp. is one of the most economically significant diseases affecting some fruits, causing fruit rot, blossom, and leaf blight which results in yield losses at both preharvest and postharvest stages [18].

Plant diseases caused by pathogenic fungi can lead to a significant reduction in plant growth potential. Therefore, this study aimed to identify total autochthonous bacterial and fungal communities associated with the ‘Williams’ variety in Serbia. Additionally, culturable bacterial and yeast communities were used to identify potential antagonistic strains in vitro originating from the pear phyllosphere effective in the suppression of phytopathogenic fungi isolated from the same variety.

## 2. Material and Methods

### 2.1. Plant Material and Culturable Microbiota

Samples of leaves and fruits were collected from the ‘Williams’ pear variety in two phenological phases—the early phases of fruit development (May) and fruit maturation (July). Samples were also collected from three independent replicates of three different trees in both phases, and every sample consisted of five leaves and five fruits. Sampling was conducted in an orchard not treated with pesticides in the last ten years, located in the Saraorci village (municipality of Smederevo, Serbia; 44°29′11″ N and 21°04′34″ E). The plant material was collected in sterile plastic bags and transported to the laboratory in a cooler. Bacterial and fungal isolation was conducted from all samples according to a procedure described previously [19]. Plant material was then covered with 200 mL of sterile phosphate-buffered saline (PBS, Sigma Aldrich, Gillingham, UK) in 1000 mL beakers and was shaken for 3 min on a rotary shaker. Washings were discarded and fresh buffer was added to plant material, sonicated for 1 min, and shaken for 15 min. For bacterial isolation, 100 µL of fresh buffer and a hundred times diluted washings were plated on Luria–Bertani (LB) agar plates and incubated for 48 h at 25 °C. A total of 100 µL of PBS washing was plated on potato dextrose agar (PDA) for fungal isolation. Additionally, whole leaves were placed on a new set of PDA plates and incubated for 7 days at 25 °C. Pure cultures of the selected bacterial isolates were maintained in LB glycerol stocks at −20 °C until further use.

### 2.2. Identification of Bacterial, Yeast, and Fungal Isolates

Total bacterial DNA was extracted as described earlier [20]. After overnight culturing in LB broth at 30 °C, bacterial cultures were centrifuged and the pellet was washed in TE buffer (10 mmol/L Tris, pH 8; 1 mmol/L EDTA). Further, the pellet was re-suspended in 500 µL of lysis buffer (50 mmol/L Tris, pH 8; 1 mmol/L EDTA, pH 8; 25% sucrose) which contained 200 µg/mL final concentration of lysozyme (Serva GMBH, Heidelberg, Germany) for DNA isolation of Gram-positive isolates. Gram-negative isolates were re-suspended in 567 µL of TE buffer with 100 µg/mL final concentration of proteinase K (Sigma, St. Louis, MO, USA) in 0.5% sodium dodecyl sulfate (SDS). After 30 min of incubation at 37 °C, 100 µL of 5M NaCl was added to the samples. Then, the samples were treated with 300 µL 3% (*w*/*v*) CTAB + PVP buffer. The mixture was incubated at 65 °C for 10 min, followed by chloroform extraction. The DNA was precipitated by cold isopropanol and ethanol and re-dissolved in 50 µL of TE buffer containing 1 µL of RNase (10 mg/mL). For the extraction of total genomic DNA from yeasts and phytopathogenic fungi, isolates were cultured on PDA plates for 48 h and seven days, respectively. From each isolate, approx. of 100 mg of yeast cells or fungal mycelia were collected and re-suspended in 200 µL of sterile water. Genomic DNA was isolated using the commercial ZR Fungal/Bacterial DNA MiniPrep^TM^ Kit according to the manufacturer’s instructions (Zymo Research, Irvine, CA, USA).

Molecular identification of bacterial isolates was performed by amplifying 16S rRNA gene sequence with universal primers UN1–16SF (5′-GAGAGTTTGATCCTGGC-3′) and UN1–16SR (5′-AGGAGGTGATCCAGCCG-3′). PCR amplifications were performed in a 25 µL reaction mixture containing 1 µg of template DNA; 25 mmol/L MgCl_2_ at the final concentration of 2.5 mmol/L; 200 mmol/L concentration of each dNTP; 1 µL of each primer; and 1 U of Taq polymerase (Fermentas UAB, Vilnius, Lithuania). The PCR reactions were performed with an initial denaturation step at 94 °C for 5 min, followed by 30 cycles of 94 °C for 30 s, 50 °C primer annealing for 1 min, and 72 °C template elongation for 30 s, followed by a final extension step at 72 °C for 7 min.

Firstly, the determination of the fungal isolates was performed on the basis of the macroscopic and microscopic characteristics. Macroscopic features include the growth appearance on PDA and against a background, as well as pigmentation of the substrate, while microscopic features entail the development of microconidia and conidiogenous cells, the presence or absence of macroconidia, chlamydospores or sclerotia, and the biometric values of the fungal reproductive organs. The standard determinants and generally accepted principles in identifying species were used, as defined by [21].

Additionally, molecular identification of antagonistic yeasts and selected micromycetes was performed by amplifying the ITS1 region with primers ITS1-F (TCCGTAGGTGAACCTGCGG) and ITS4-R (TCCTCCGCTTATTGATATGC) [22], and β-tubulin gene with T1-F (AACATGCGTGAGATTGTAAGT) and BT12-R (GTTGTCAATGCAGAAGGTCTCG) primers [23,24]. The PCR reactions were performed as follows: one denaturation cycle at 95 °C for 10 min, followed by 30 cycles of denaturation at 95 °C for 30 s, annealing at 55 °C for 45 s, and extension at 72 °C for 90 s, with one final cycle of extension at 72 °C for 10 min. All PCR amplicons were purified using the QIAquick PCR Purification Kit according to the manufacturer’s protocol (Qiagen, Hilden, Germany) and later sequenced by Macrogen, Inc. (Amsterdam, the Netherlands) using the same primers as for amplification.

Obtained sequences were used to search for homology with sequenced genes in the GenBank database with the National Center for Biotechnology Information (NCBI) BLASTN program and all chromatograms were checked manually. All sequences were aligned with reference strain sequences from the GenBank database using CLUSTAL W implemented in BioEdit 7.2.6 software, while phylogenetic trees were constructed in MEGA X software using the neighbor-joining method based on a pairwise distance matrix with the Kimura two-parameter nucleotide substitution model.

### 2.3. Antifungal Activity of Autochthonous Bacterial and Yeast Isolates

The potential antifungal activity of 18 bacterial and 6 yeast isolates from the pear phyllosphere was tested in vitro against ten potential pears’ preharvest and postharvest phytopathogenic fungi, obtained from the same phyllosphere in the same orchard. The bacterial strains were cultured overnight in LB broth at a temperature of 30 °C. The yeast isolates were cultured under the same conditions in yeast dextrose broth (YDB). For initial screening of their antagonistic activity, PDA plates in rectangular form were inoculated with broth culture about 2.5 cm away from a seven-day-old fungal mycelial plug. After seven days of incubation at 25 °C, antifungal activity was observed. For further screening of antagonistic activity, the cells of the best candidates were harvested by centrifugation at 13,000× *g* for 15 min, and the culture supernatant was filtered through 0.45 μm Durapore^TM^ (Millipore, Billerica, MA, USA) filters. An amount of 100 µL of supernatants was spread on a PDA plate and a mycelial plug from the margin of seven-day-old cultures was placed in the center of the plate. Plates inoculated with only fungal isolates were used as controls. Inoculated plates were incubated for 7 days at 25 °C. The effect of supernatants on mycelial growth was obtained by calculating the percentage inhibition of radial growth using the formula $\;PIRG\%=\frac{100\left(KR-R1\right)}{KR}$, where KR represents the diameter of mycelial growth fungus in the control plates, and R1 is a growth of the test fungus in the presence of the bacterial/yeast supernatant. The experiments were repeated twice independently, with three replications for each fungus.

The analysis of variance was supported by the Kolmogorov–Smirnov test for the normality of residuals and obtained data were subjected to the variance analysis (one-way ANOVA). The means separation was accomplished by Tukey’s HSD test with a significance level of *p* < 0.05. Statistical analyses were conducted using general procedures of STATISTICA ver. 7 (StatSoft Inc., Tulsa, OK, USA) and IBM SPSS Statistics v.20 (IBM SPSS Inc., Armonk, NY, USA).

### 2.4. Amplicon Sequencing of Unculturable Microbiota

The same samples prepared for isolation of culturable microbiota were also used for metabarcoding analysis. Total DNA from each sample from three individual trees was isolated and pooled into one sample for both phenophases. The plant material of each sample was washed with 1× PBS and washings were used for the extraction of total DNA from the pear phyllosphere. For each sample, 100 mL of PBS from plant material washing was filtered with Isopore^TM^ membrane filters (Merck Millipore Ltd., Carrigtwohil, Ireland). The DNA was extracted from 0.22-µm polycarbonate Isopore^TM^ filters using the ZymoBIOMICS^TM^ DNA Mini Kit (Zymo Research, Irvine, CA, USA) following the manufacturer’s instructions. Qubit fluorometric quantitation (Qubit 4 fluorometer, Invitrogen™, Waltham, MA, USA) was used for the quantification of isolated DNA. Further, the DNA samples were dissolved in DNase/RNase-free water and commercially sequenced (Fisabio, Valencia, Spain) using a 2 × 300-bp paired-end run on a MiSeq Sequencer, according to the manufacturer’s instructions (Illumina, San Diego, CA, USA). The 16S rRNA gene-specific sequences to target the V3 and V4 regions were used in this study, with the defined forward (5′-CCTACGGGNGGCWGCAG-3′) and reverse (5′-GACTACHVGGGTATCTAATCC-3′) primers [25]. To target the internal transcribed spacer (ITS) regions, the forward ITS1 KYO2 (5′-TAGAGGAAGTAAAAGTCGTAA-3′), and reverse ITS2_KYO2 (5′-TTYRCTRCGTTCTTCATC-3′) primers were used [26].

### 2.5. Reprocessing, Sequence Inference, Taxonomy Annotation, and Data Availability

Quality assessment and sequence trimming was conducted using literal primer sequences as stated above with a k-mer length of 15 and Hamming distance of 1 was performed using the BBduk software package [27]. All sequences that had more than 3 for forward and 2 for reverse strand expected errors (calculated as the sum(10^(-Q/10))—where Q is the quality score), were discarded (argument: maxEE = c(3, 2)), as well as sequences shorter than 50 bp. Additionally, a sequence merger with a minimum overlap of 20 bases without mismatches was performed. Data analysis was conducted using an ad hoc DADA2 pipeline for denoising, paired-end joining, and chimera depletion, starting from the paired-ends data [28]. All sequences with a length shorter than 280 bp and longer than 367 bp for ITS, as well as shorter than 400 bp and longer than 427 bp for 16S were removed, respectively. Taxonomic affiliations were assigned using the RDP naive Bayesian classifier [29] with taxonomy assignment to the SILVA 138 for 16S (https://www.arb-silva.de/documentation/release-138/, accessed on 4 February 2022) and UNITE (https://plutof.ut.ee/#/doi/10.15156/BIO/786368, accessed on 4 February 2022) for ITS sequences. In addition, ASVs with high abundance and ambiguous taxonomy assignments were annotated based on the BLAST best hit in the National Center for Biotechnology Information (NCBI) nucleotide database up to the species level of annotation. The main diversity data reported were obtained by considering the whole dataset, including singletons and/or under-represented taxa, except the sequences assigned to chloroplasts and mitochondria, which were excluded from further analysis.

Sequence diversity within samples (alpha diversity) was estimated using the phyloseq R package [30] at the amplicon sequence variant (ASV), genus, family, and phylum levels. Alpha diversity of fungal and bacterial communities was shown through estimators Shannon and Simpson. Observed and estimated richness was determined according to the following estimators: a number of observations (OBS), Chao1, and ACE. Prior to the computation of the diversities and distances, samples were rarefied to even depth according to the sample with the lowest number of reads [31].

All data were deposited within the NCBI database as BioProject ID: PRJNA843330.

## 3. Results

### 3.1. Culturable Microbiota

**Figure 1:**
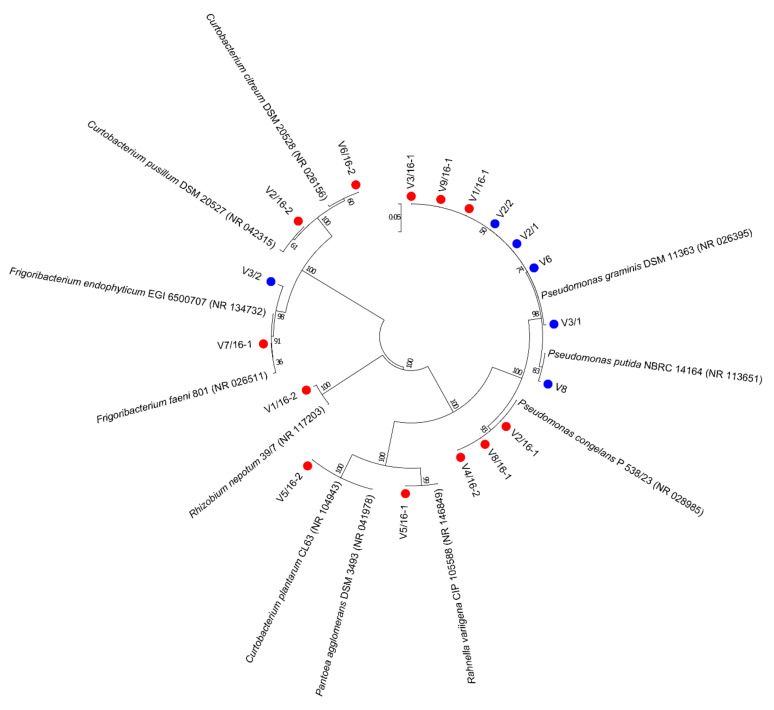
Phylogenetic relationships of bacterial isolates from ‘Williams’ pear variety in two phenological stages (May ● and July ●) based on the 16S rRNA sequence. A phylogenetic tree was constructed by the Neighbor-joining method and the distances were calculated with the Kimura two-parameter model. Bootstrap values are given for each node, with 1000 replicates.

During two phenological phases of the ‘Williams’ pear variety, from collected samples of fruit and leaves, a total of 18 bacterial isolates were obtained. The analysis of the 16S rRNA sequences indicated the presence of six genera and eight species. The most abundant were isolates from the *Pseudomonas* genus identified as *P. graminis*, *P. putida*, and *P. congelans*. Other bacterial isolates were identified as representatives of genera *Pantoea, Rhizobium, Curtobacterium, Rahnella*, and *Frigoribacterium*. Identified genera differed as a result of sampling times, in the spring samples (May) only *Pseudomonas* and *Frigoribacterium* were isolated whilst in the summer samples (July) isolates from all 6 identified genera were identified. Only the isolates of *P. graminis* and *F. endophyticum/faeni* were identified in both sampling times. Phylogenetic relationships of identified species are shown in Figure 1.

According to the macroscopic and microscopic characteristics of the fungal growth, a total of 28 isolates were obtained. The number of unique species for both phenological stages was different and most of them were isolated in May, while for both stages ten isolates were common (Table 1). The most abundant were representatives from the genus *Fusarium* with six identified species (*F. incarnatum, F. verticillioides, F. proliferatum, F. oxysporum, F. solani*, and *F. sporotrichioides*). More than one detected species was observed for genera *Trichoderma, Phoma*, and *Aspergillus.*

**Figure 2:**
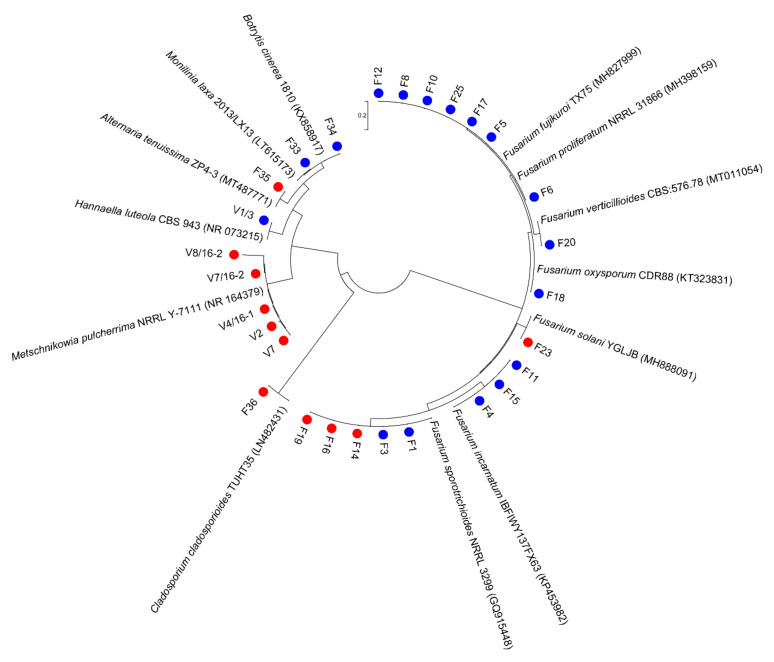
Phylogenetic relationships of fungal isolates from the ‘Williams’ pear variety in two phenological stages (May ● and July ●). The phylogenetic tree was constructed by the maximum likelihood method using the Kimura two-parameter model.

Sequences obtained by amplifying the ITS region were used for the identification of yeast isolates, while the ITS/β-tubulin combination was used for the purpose of identifying the selected isolates tested in the further assay. *Metschnikowia pulcherrima* was dominantly present with five cultivated isolates only in the spring samples (May), while *Hannaella luteola* was characteristic for the summer samples (July). All other fungal isolates were confirmed by molecular identification and their phylogenetic relationships are presented in Figure 2.

### 3.2. Antifungal Activity

Initial screening of antagonistic activity by beneficial bacteria from risk group 1 and yeasts (Figure S1) was performed against preharvest (*Monilinia laxa*, *Botrytis cinerea*, *Alternaria tenuissima*, and *Cladosporium cladosporioides*) and postharvest pathogens from the *Fusarium* genus (*F*. *proliferatum*/*fujikuroi*, *F*. *verticillioides*, *F. sporotrichioides*, *F. solani*, *F. oxysporum*, and *F. incarnatum*). Antifungal activity tested in a dual culture on PDA plates confirmed five bacterial and three yeast isolates, among others, and they were selected for further tests. The supernatants of the selected cultures grown overnight were further tested against the same pathogens (Table 2).

*Fusarium sporotrichioides* was detected as the most susceptible fungal isolate, with high percentages of growth inhibition, with most antagonists in the range of 53–70% of all tested antagonistic strains. Contrarily, *F*. *verticillioides*, *F. oxysporum*, and *F. incarnatum* were the most resistant isolates since only the fewest antagonists were effective against them. Among the tested antagonistic isolates, the *Hannaella luteola* V1/3 and *Metschnikowia pulcherrima* V7 yeasts showed a broad range of the strongest activity, significantly affecting the growth of many fungal isolates with a more than 50% of inhibition rate (up to more than 70% in the case of *H. luteola* activity). *Hannaella luteola* significantly inhibited the growth of about nine fungi, while *F*. *endophyticum/faeni* showed the weakest antifungal activity. Apart from yeasts, *Pseudomonas graminis* V2/1 might be a potentially good antagonistic strain with a moderate, but broad range of significant activity.

### 3.3. Metabarcoding Data and Alpha Diversity of Microbial Communities

Alpha diversity and phylogenetic composition of the pear microbiome were analyzed using pooled DNA samples isolated from the ‘Williams’ variety in two phenological early phases of fruit development (May, TJV1) and fruit maturation (July, TJV2). In order to characterize the bacterial community, the V3-V4 region of 16S rRNA was sequenced. After amplifying, trimming, and quality filtering the reads, 111,369 (May) and 106,836 (July) paired-end sequence reads were retained. Following the chimera-checking steps and chloroplast/mitochondria DNA removal, total numbers of 644 and 432 ASVs were obtained in May and July. The fungal communities were characterized by a sequencing of the ITS1 region from the same DNA samples. Amplified reads were recovered from 178,901 high-quality sequences in May (assigned to 285 ASV), and 164,796 in July (276 assigned ASVs). The microbial richness and alpha diversity indices on the phylum, family, genus, and ASV (the amplicon sequence variant) level are presented in Table 3. Microbial communities were rich and diverse in both sampling periods, with a higher prevalence of bacterial taxa than fungal communities. Higher values of observed and estimated bacterial richness were detected in May at all observed taxonomic levels compared with samples from July. A similar trend was detected for richness and diversity indices for fungal communities in May and July, especially according to the Shannon index.

### 3.4. The Composition of the Pear’S Bacteriota

**Figure 3:**
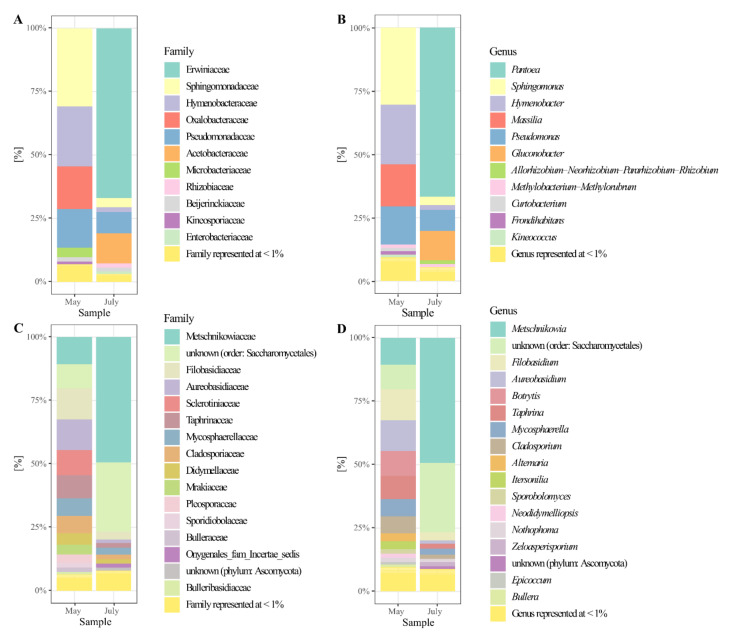
Relative abundance (RA) of bacterial and fungal families (**A**,**C**) and genera (**B**,**D**) associated with the ‘Williams’ pear variety at two phenological stages (May and July). Identified taxa above 1% of the total RA are presented.

According to the analysis of the 16S rRNA gene sequences at the phylum level, Proteobacteria was the most abundant at both sampling time points, with 67% in May and 97% in July. A higher abundance of the phylum Bacteroidetes, about 24%, was detected in May samples (data are not shown). A relative abundance of the family and genera with a normalized abundance of over 1% are presented in Figure 3A,B. In the early phase of the fruit development (May), at the family level, the most abundant representatives were identified among Sphingomonadaceae (31%), Hymenobacteraceae (23%), Oxalobacteraceae (17%), and Pseudomonadaceae (15%).

**Figure 4:**
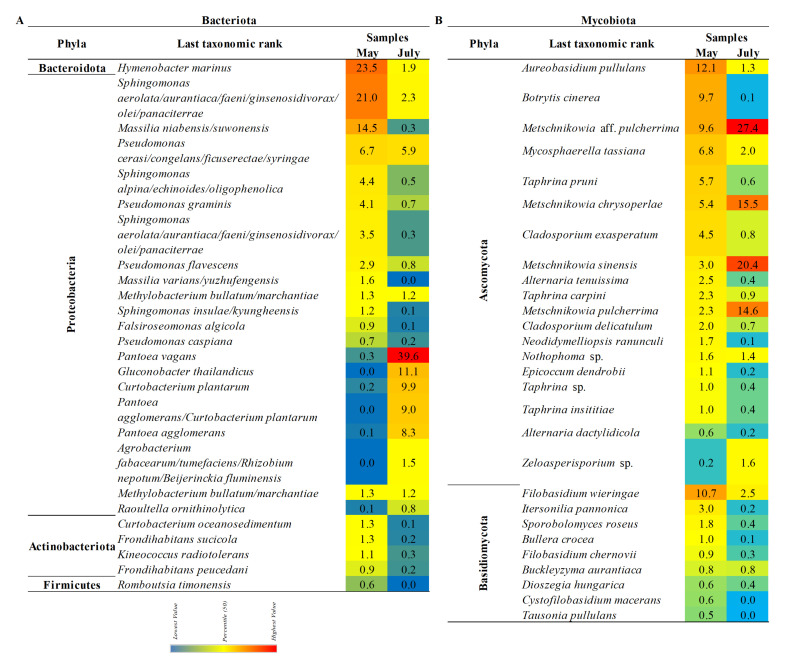
Relative abundance (RA) of bacterial (**A**) and fungal (**B**) species associated with the ‘Williams’ pear variety at two phenological stages. Species with a total RA above 0.5% are presented.

In the early phase of the fruit maturation (July) the dominant presence of the family Erwiniaceae (67%) was observed. Acetobacteraceae had a significant abundance (12%), compared with May samples when their presence wasn’t detected above 1%. Contrarily, the abundance of Pseudomonadaceae representatives decreased (9%) compared with May samples. On the genus and species level, most prevalent in the May samples were representatives of *Sphingomonas* (30%) with four different species: *Hymenobacter marinus* (23%), *Massilia* (16%) with two abundant species (*M. niabensis/suwonensis* and M. *varians/yuzhufengensis*), and *Pseudomonas* (15%) with four species, identified as *P. flavescens*, *P. caspiana*, *P. graminis*, and *P. cerasi/congelans/ficuserectae/syringae* (Figure 4A). Later in the season, the most abundant were *Pantoea* (67%), with two identified species *P. vagans* and *P. agglomerans*, and *Gluconobacter thailandicus* (11%), while the abundance of the *Pseudomonas* (8%), and *Sphingomonas* (3%) genera decreased.

### 3.5. The Composition of the Pear’s Mycobiota

Phylum Ascomycota was dominantly present in the pear phyllosphere with a detected abundance of 75% in May and 92% in July. The significant presence of phylum Basidiomycota (24%) was also scored for the May samples. Relative abundance of the families and genera with a normalized abundance of over 1% is presented in Figure 3C,D. On the family level, the most abundant were representatives of Filobasidiaceae (12%), Aureobasidiaceae (12%), Metschnikowiaceae (11%), Sclerotiniaceae (10%), an unidentified family within the order Saccharomycetales (9%), Taphrinaceae (9%), Mycosphaerellaceae (7%), and Cladosporiaceae (7%) in May. In July, the composition of the fungal community changed, when the dominant presence of representatives from Metschnikowiaceae (49%) and Saccharomycetales (27%) was detected. Hence, a change in the abundance of corresponding genera was also observed from May through July.

In May, the most abundant were representatives of the genera and species including *Filobasidium* (12%) with two identified species *F. wieringae* and *F. chernovii, Aureobasidium pullulans* (12%)*, Metschnikowia* (20%)*, Botrytis cinerea* (10%)*, Taphrina* (9%) with three identified species, followed by *Mycosphaerella tassiana* (7%)*, Cladosporium exasperatum* and *C. delicatulum* (7%), *Alternaria tenuissima* and *A. dactylidicola* (3%), and *Itersonilia pannonica* (3%). In July, the assessed diversity was decreased with only four genera detected with an abundance of over 2% including *Metschnikowia* (76%), *Filobasidium* (12%), and *Mycosphaerella* (7%). *Metschnikowia* aff. *Pulcherrima, M. sinensis*, and *M. chrysoperlae* were identified as the most prevalent species (Figure 4B).

## 4. Discussion

The present study was the first to evaluate the diversity of bacterial and fungal communities associated with the phyllosphere (fruits and leaves) of the ‘Williams’ pear variety. Both bacterial and fungal communities were rich in diversity within the fruit development and maturation phases. Variation in diversity and richness was detected between sampling points, with decreased alpha diversity indices in the July samples. The abundance and diversity of detected taxa have changed during two sampling periods indicating that changes occurred during the maturation of the leaves and fruit. Furthermore, environmental factors could also be responsible for affecting the composition of the microbiota. Earlier studies also reported seasonal influence on the richness and diversity of the fungal and bacterial communities associated with the phyllosphere [19,20,32,33].

Phyla Proteobacteria and Bacteroidetes are one of the most common groups inhabiting the phyllosphere of different plant hosts. Smessaert et al. [7] showed that Proteobacteria was one of the most prevalent members of the floral nectar microbiota on the ‘Conference’ pear, which also includes the moderate presence of Bacteriodetes representatives, which is in accordance with our study. Members of the Alphaproteobacteria were earlier identified as predominant and ubiquitous in phyllosphere microbiota. Within the same class, the genus *Sphingomonas* is consistently detected among different hosts [34]. Species of the *Sphingomonas* spp. have been found able to reduce symptoms of plant diseases and suppress pathogen populations by showing a protective effect on the host plant. This trait has been recognized in *Sphingomonas* spp. when isolated from plants but not from other habitats [35]. Besides *Sphingomonas* spp., other bacterial genera detected with high abundance on the ‘Williams’ variety were also commonly detected as residents of the plant phyllosphere including *Pseudomonas*, *Pantoea*, and *Massilia*. Their representatives have beneficial effects on hosts by protecting them from diseases, as well as promoting their growth by different mechanisms [35,36]. As in our previous study on bacterial communities of plum phyllosphere conducted in the same organic orchard, the dominance of the *Sphingomonas, Pseudomonas*, and *Hymenobacter* spp. was also observed [20]. Recently, Luziatelli et al. [37] showed the beneficial effects of the metabolites secreted by a plant-growth-promoting *Pantoea agglomerans* on the *Pyrus communis* variety ‘Dar Gazi’ by improving its rooting. Considering the effect of the fruit phenophase on the composition of culturable microbiota, Janisiewicz et al. [38] also observed a decline in the abundance of *Pseudomonas* spp. isolates during fruit maturation. In their study, *Pantoea* spp. increased as nectarine fruit maturated. Although little is known about the role of the genus *Hymenobacter* in the phyllosphere, several studies identified them as common phyllosphere residents [20,39,40,41]. In addition to common species characteristics for phyllosphere microbiota, we also observed a high abundance of *Gluconobacter* spp. when ‘Williams’ fruits were in the early phase of maturation. Meanwhile, some of the most abundant bacterial genera declined, which indicated changes in microbiota composition related to fruit maturation. For instance, Zhang et al. [42] found a negative correlation pattern between *Gluconobacter* and *Sphingomonas*, which may be due to the high abundance of *Gluconobacter* that potentially reduced the growth of other microorganisms. This genus is a member of the family Acetobacteraceae, comprised of acetic acid bacteria, and it is capable of causing fruit decay in apples and pears. Also, *Gluconobacter* strains were found to be very abundant in sugary niches in different ripe fruits [43], thus indicating how changes in the fruit content during maturation potentially affected its high prevalence in the ‘Williams’ pear microbiota. Nevertheless, Bevardi et al. [44] reported the ability of the *Gluconobacter oxydans*, isolated from the apple surface, to antagonize *Penicillium expansum*, the pathogen that causes post-harvest fruit decay. Also, Zhang et al. [42] indicated *Gluconobacter* sp. as an antagonistic or saprophytic bacterium related to the surface of pears.

Analyzing the composition of the fungal community of the ‘Williams’ phyllosphere, revealed that the most abundant phylum was Ascomycota, including associated families. Previous studies also detected Ascomycota as dominant in phyllosphere microbiota [45]. A notable presence of yeasts was detected in both analyzed phases of fruit development, including *Aureobasidium, Filobasidium*, and *Metschnikowia*. *Filobasidium* spp. is a basidiomycetous yeast often found in the phyllosphere of different hosts, such as grapes, barley, and melon [46,47,48]. *Filobasidium wieringae* was identified as predominant in the epiphytic community of pear fruit [49] which coincides with our results. *Aureobasidium pullulans* and different *Metschnikowia* spp. species are well known for biocontrol potential in disease suppression caused by important pathogens like *Botrytis cinerea, Monilinia laxa, M. fructicola*, and *M. fructigena* [50,51,52,53,54]. The abundance of *M. sinensis* and *M. chrysoperlae* rapidly increased in the July samples on the ‘Williams’ variety. This occurred simultaneously with the reduction of pathogens from genera *Botrytis*, *Taphrina*, and *Alternaria*. The biocontrol potential of *Metschnikowia* spp. could be a reason for this beneficial shift in the composition of pear mycobiota. The same trend was noticed in our previous study analyzing the fungal community of the plum phyllosphere [19]. Furthermore, during maturation, when micro damages on the cuticle and softening of the fruit occur, the availability of nutrients, including sugar content, increases. This may explain the dominance of fermentative yeasts of the genus *Metschnikowia* [55]. Glushakova et al. [49] reported an increase in the number of epiphytic yeasts on the *Pyrus communis* fruit surface during ripening, characterized by maximum sugar exudation on the fruit surface, and a soft and damaged cuticle. Furthermore, the non-Saccharomyces yeasts *Aureobasidium*, as well as *Metschnikowia* play an important role in spontaneous alcoholic fermentations and influence the quality of the composition and aroma of alcoholic beverages [56].

Pathogenic representatives including *Botrytis, Taphrina*, and *Alternaria* are commonly found in different crops [57,58,59]. Most of the reads associated with *Botrytis* spp. were identified on the species level as *B. cinerea*, the causative agent of gray mold, a destructive fungal disease of a wide range of crops [58]. This pathogen causes calyx-end decay on pears, a disease that results in considerable economic losses [60]. On the other hand, *Cladosporium* species are cosmopolitan, agents of plant decay, common endophytes, and phylloplane fungi [61]. Our study on the ‘Williams’ phyllosphere identified, using amplicon sequencing, mostly saprophytic representatives, including *C. delicatulum, Mycosphaerella tassiana* as a teleomorph of *C. herbarum*, and *C. exasperatum*. *Cladosporium delicatulum* was earlier reported as a mycoparasite of *Taphrina pruni* [62], but interaction is beneficial for plant hosts since the mycoparasitism is recognized as one of the biocontrol strategies [63]. However, the culturable approach provided us with the *C. cladosporioides* isolate, which has been proven together with the *C. herbarum* complex species to cause lesions in healthy pears [64]. We also identified *Itersonilia pannonica*, the yeast found to be a ubiquitous colonizer of plant tissues [65]. A community metabarcoding study by Rojas et al. [66] revealed *C. herbarum* and *I. pannonica* as beneficial endophytes able to suppress the *Fusarium* blight disease on wheat and to keep wheat spikes healthy despite exposure to the pathogen.

In addition to characterizing the total microbiota of the ‘Williams’ pear phyllosphere, we also conducted isolation of bacteria and yeasts from the same samples as potential candidates for biological control of different fungi. *Pseudomonas* species were the most represented among culturable microbiota, and were detected in both analyzed phenological phases. Duvenage et al. [67] also detected *Pseudomonas, Pantoea*, *Curtobacterium*, and *Frigoribacterium* spp. as common culturable inhabitants on freshly harvested pear fruit of the ‘Packham’s Triumph’ variety. As was earlier discussed, these species have potentially beneficial effects on plant welfare. In the ‘Williams’ phyllosphere, we also detected the recently described species, *Rahnella variigena*, which was isolated from oak as an endophyte [68]. Among culturable yeasts, *Metschnikowia pulcherrima*, which is well known as a resident of the fruit phyllosphere [69], and *Hannaella luteola*, earlier isolated from wine grapes [70], were also found in our study. Apart from beneficial bacteria, we also found *Rhizobium nepotum*, which was previously detected as a causative agent of crown gall on different *Prunus* species [71].

After the initial screening was conducted to identify isolates with strong antagonistic activity, the isolate V1/3 identified as *H. luteola* showed the strongest antifungal activity against almost all tested isolates. To date, *H. luteola* has not been identified as a biocontrol agent. Many yeast species have been reported to be capable of controlling plant-pathogenic fungi by diverse mechanisms, including secretion of cell-wall lytic enzymes and siderophores, the release of volatile compounds, etc. [72]. In addition to *H. luteola*, the isolate of *M. pulcherrima* had a broad range of activity against all tested fungal strains. *Metschnikowia pulcherrima* has been established as highly efficient against *M. laxa*, the causative agent of brown rot [73]. *M. pulcherrima* was also found to have great potential for controlling *F. fujikuroi* on the rice crop [74]. The toxin-producing *Fusarium* species, including *F. sporotrichioides*, can induce significant yield losses in affected crops and accumulated mycotoxins are dangerous to human and animal health [75]. Additionally, it has been shown that some *Fusarium* species are able to cause yeast-like fermentative fruit spoilage [6], while a recently described case of *Fusarium avenaceum* was seen to be a causative agent of branch canker on pears in Turkey [76]. Therefore, finding eco-friendly biocontrol agents for suppressing these mycotoxin producers is a suitable strategy to both reduce the application of chemical fungicides and prevent the accumulation of harmful toxins in the different crops. We observed that *H. luteola*, *M. pulcherrima*, and *P. graminis* were efficient to both *F. sporotrichioides* and *M. laxa* so they could, in the future, be considered as a microbial consortium for the treatment of these important phytopathogens. This is in accordance with and earlier hypothesis that established that the phyllosphere is the best source of effective biocontrol agents [77].

In conclusion, this is the first study that analyzed the fungal and bacterial diversity at two stages of the ‘Williams’ fruit development, using combined approaches. Our results demonstrated important differences in microbiota diversity during the maturation of pear fruit. Furthermore, culturable communities from the pear phyllosphere were shown to be an important source of antagonistic microorganisms. *Hannaella luteola*, to the best of our knowledge, was identified as a potential biocontrol agent for the first time. Additionally, strains of *H. luteola, M. pulcherrima*, and *P. graminis* stood out as antagonists with the potential to be considered in the future as a consortium for the treatment of many phytopathogenic fungi.

## Figures and Tables

**Table 1:** Fungal isolates from ‘Williams’ pear variety’s phyllosphere.

Phenophases	Total Number of Species	Identified Species
May	13	*Phoma* sp., *Nigrospora* sp., *Trichoderma harzianum*, *Penicillium* sp., *Botrytis cinerea*, *Pseudopithomyces* sp., *Fusarium incarnatum*, *Phoma putaminum*, *Epicoccum* sp., *Trichothecium roseum*, *Macrophomina phaseolina, Fusarium verticillioides, Fusarium proliferatum*
July	5	*Penicillium corylophilum*, *Alternaria tenuissima*, *Cladosporium cladosporioides*, *Stemphylium botryosum*, *Curvularia* sp.
May and July	10	*Aspergillus niger, Aspergillus flavus, Trichoderma viride, Alternaria* sp., *Fusarium oxysporum, Fusarium solani, Cladosporium* sp., *Phoma betae, Fusarium sporotrichioides, Rhizopus* sp.

**Table 2:** Antifungal activity (mean ± SE) of supernatants from the antagonistic isolates on selected fungi in vitro, by a method of dual cultivation. The percentage inhibition of radial growth (PIRG%) as a measure of the antifungal activity was calculated.

**Antagonists**	*Fusarium proliferatum*/*Fujikuroi*	*Fusarium verticillioides*	*Fusarium sporotrichioides*	*Fusarium solani*	*Fusarium oxysporum*	*Fusarium incarnatum*
Bacteria						
*Pseudomonas graminis* V2/1	**39.8 ^a^ ± 2.00**	39.0 ^ab^ ± 1.99	65.1 ^ab^ ± 1.04	28.0 ^bc^ ± 3.18	42.5 ^ab^ ± 1.88	21.6 ^c^ ± 6.43
*Pseudomonas graminis* V2/2	11.4 ^cd^ ± 2.40	11.7 ^e^ ± 0.75	**69.4 ^a^ ± 1.80**	12.6 ^cd^ ± 1.46	13.0 ^cd^ ± 0.50	16.1 ^c^ ± 2.63
*Pseudomonas graminis* V3/1	12.6 ^bcd^ ± 0.84	19.9 ^de^ ± 2.41	52.9 ^b^ ± 1.36	10.9 ^cd^ ± 1.57	18.9 ^cd^ ± 2.00	8.8 ^c^ ± 3.13
*Pseudomonas putida* V8	19.3 ^bc^ ± 2.31	9.1 ^e^ ± 1.50	65.5 ^ab^ ± 2.08	3.5 ^d^ ± 1.10	7.4 ^d^ ± 2.29	6.4 ^c^ ± 2.04
*Frigoribacterium endophyticum/faeni* V3/2	4.5 ^d^ ± 0.80	15.2 ^de^ ± 2.29	65.1 ^ab^ ± 3.42	0.0 ^d^ ± 0.00	14.6 ^cd^ ± 2.13	15.7 ^c^ ± 2.03
Yeasts						
*Metschnikowia pulcherrima* V2	21.6 ^b^ ± 2.01	22.9 ^cd^ ± 3.03	63.1 ^ab^ ± 5.28	46.3 ^ab^ ± 4.76	24.8 ^bcd^ ± 2.67	27.3 ^bc^ ± 6.22
*Metschnikowia pulcherrima* V7	**45.6 ^a^ ± 2.44**	33.6 ^bc^ ± 3.00	52.7 ^b^ ± 5.96	**52.4 ^a^ ± 2.34**	26.4 ^bc^ ± 7.95	50.9 ^ab^ ± 3.21
*Hannaella luteola* V1/3	**42.3 ^a^ ± 2.01**	**45.5 ^a^ ± 1.98**	**70.2 ^a^ ± 1.03**	**56.9 ^a^ ± 8.56**	**53.2 ^a^ ± 1.98**	**56.9 ^a^ ± 8.56**
	*Monilinia laxa*	*Botrytis cinerea*	*Alternaria tenuissima*	*Cladosporium cladosporioides*		
Bacteria						
*Pseudomonas graminis* V2/1	**63.2 ^a^ ± 0.44**	42.6 ^ab^ ± 1.29	40.5 ^bc^ ± 0.88	52.2 ^ab^ ± 2.51		
*Pseudomonas graminis* V2/2	0.0 ^b^ ± 0.00	35.4 ^bc^ ± 8.31	34.4 ^c^ ± 0.88	43.4 ^ab^ ± 2.51		
*Pseudomonas graminis* V3/1	17.8 ^b^ ± 1.49	36.6 ^bc^ ± 8.75	43.6 ^b^ ± 2.64	45.6 ^ab^ ± 6.26		
*Pseudomonas putida* V8	15.7 ^b^ ± 3.46	38.5 ^ab^ ± 3.97	34.4 ^c^ ± 0.88	14.4 ^c^ ± 1.43		
*Frigoribacterium endophyticum/faeni* V3/2	5.7 ^b^ ± 1.54	12.8 ^c^ ± 3.44	21.7 ^d^ ± 1.02	36.9 ^b^ ± 6.26		
Yeasts						
*Metschnikowia pulcherrima* V2	**52.2 ^a^ ± 0.77**	46.3 ^ab^ ± 3.86	**60.5 ^a^ ± 1.50**	52.2 ^ab^ ± 2.51		
*Metschnikowia pulcherrima* V7	**54.0 ^a^ ± 6.92**	**61.7 ^a^ ± 2.56**	**64.9 ^a^ ± 0.88**	**58.0 ^a^ ± 1.47**		
*Hannaella luteola* V1/3	**65.0 ^a^ ± 6.43**	45.5 ^ab^ ± 2.22	**65.8 ^a^ ± 3.03**	**60.9 ^a^ ± 2.51**		

Mean values followed by the same superscript letter within columns are not significantly different (*p* < 0.05), according to Tukey’s HSD test. The values in **bold** represent the highest percent of statistically significant inhibition for a particular fungus as a result of the action of a certain isolate’s supernatant.

**Table 3:** Bacterial and fungal community richness and diversity of the ‘Williams’ pear variety at two phenological stages presented through alpha diversity indices at the phylum, family, genus, and ASV level. Richness is estimated by observed (OBS), Chao1, and ACE estimators, while diversity was evaluated by Shannon and Simpson indices.

16S									
Sample	OBS	Chao1	SE.Chao1	ACE	SE.ACE	Shannon	Simpson	InvSimpson	Level
May	12.00	12.00	0.00	12.00	1.71	0.90	0.48	1.93	Phylum
July	8.00	8.00	0.00	8.00	1.37	0.16	0.06	1.06	
May	61.00	61.00	0.00	61.00	3.89	1.90	0.80	4.88	Family
July	32.00	32.00	0.00	32.00	2.62	1.28	0.53	2.12	
May	99.00	99.08	0.34	99.67	4.94	2.05	0.80	5.02	Genus
July	66.00	66.00	0.08	66.41	3.38	1.37	0.53	2.14	
May	644.00	694.04	14.44	693.57	12.86	4.23	0.94	17.29	ASV
July	432.00	454.00	8.83	456.30	10.55	2.48	0.80	5.06	
**ITS**									
**Sample**	**OBS**	**Chao1**	**SE.Chao1**	**ACE**	**SE.ACE**	**Shannon**	**Simpson**	**InvSimpson**	**Level**
May	3.00	3.00	0.00	–	–	0.59	0.38	1.61	Phylum
July	3.00	3.00	0.00	–	–	0.29	0.15	1.18	Phylum
May	72.00	72.00	0.00	72.00	3.26	2.73	0.92	11.99	Family
July	67.00	67.00	0.00	67.00	2.92	1.76	0.68	3.11	
May	109.00	109.00	0.00	109.00	3.79	2.86	0.92	12.39	Genus
July	93.00	93.00	0.00	93.00	3.55	1.81	0.68	3.11	
May	285.00	285.00	0.00	285.00	5.86	3.78	0.95	20.81	ASV
July	276.00	276.00	0.00	276.00	5.46	4.27	0.97	39.68	

## Data Availability

The data presented in this study are openly available in [NCBI] at [https://www.ncbi.nlm.nih.gov/bioproject?term=PRJNA843330&cmd=DetailsSearch], accessed on 4 February 2022, as BioProject ID: PRJNA843330.

## Supplementary Materials

The following supporting information can be downloaded at: https://www.mdpi.com/article/10.3390/microorganisms10071282/s1, Figure S1: Dual culture method for determining the antagonistic potential of bacterial and yeast isolates. The activity of selected isolates against: *Botrytis cinerea* (A—control, B—treatment). *Cladosporium cladosporioides* (C—control, D—treatment), *Alternaria tenuissima* (E—control, F—treatment), and activity of *Hannaella luteola* V1/3 supernatant against *B. cinerea* (G—control, H—treatment) were shown.
