# Supplementary material for: The Microbiome of the ‘Williams’ Pear Variety Grown in the Organic Orchard and Antifungal Activity by the Autochthonous Bacterial and Yeast Isolates
Source: Microorganisms. 2022 Jun 24;10(7):1282. doi: 10.3390/microorganisms10071282 (PMC9321879; doi:10.3390/microorganisms10071282)
Supplement: Supplementary file 1 [file microorganisms-10-01282-s001.zip › microorganisms-1776496-supplementary.pdf]

Supplementary Materials

The Microbiome of the 'Williams' Pear Variety Grown in the Organic Orchard and Antifungal Activity by the Autochthonous Bacterial and Yeast Isolates

Tamara Janakiev [1], Tanja Berić [1], Tatjana Stević [2], Slaviša Stanković [1], Jasmina Bačić [3], Helena Majstorović [3], Djordje Fira [1] and Ivica Dimkić [1,*]

[1] Faculty of Biology, University of Belgrade, Studentski trg 16, 11158 Belgrade, Serbia; tamara.janakiev@bio.bg.ac.rs (T.J.); tanjab@bio.bg.ac.rs (T.B.); slavisas@bio.bg.ac.rs (S.S.); fira@bio.bg.ac.rs (D.F.)

[2] Institute for Medicinal Plants Research "Dr. Josif Pančić", Tadeuša Košćuška 1, 11000 Belgrade, Serbia; tanjasomosa@gmail.com

[3] PSS "Tamiš" Institute, Agriculture Extension Service Province of Vojvodina, Novoseljanski put 33, 26000 Pančevo, Serbia; jasminabacic4691@gmail.com (J.B.); majstorovic@institut-tamis.rs (H.M.)

* Correspondence: ivicad@bio.bg.ac.rs; Tel./Fax: +381-11-2637364

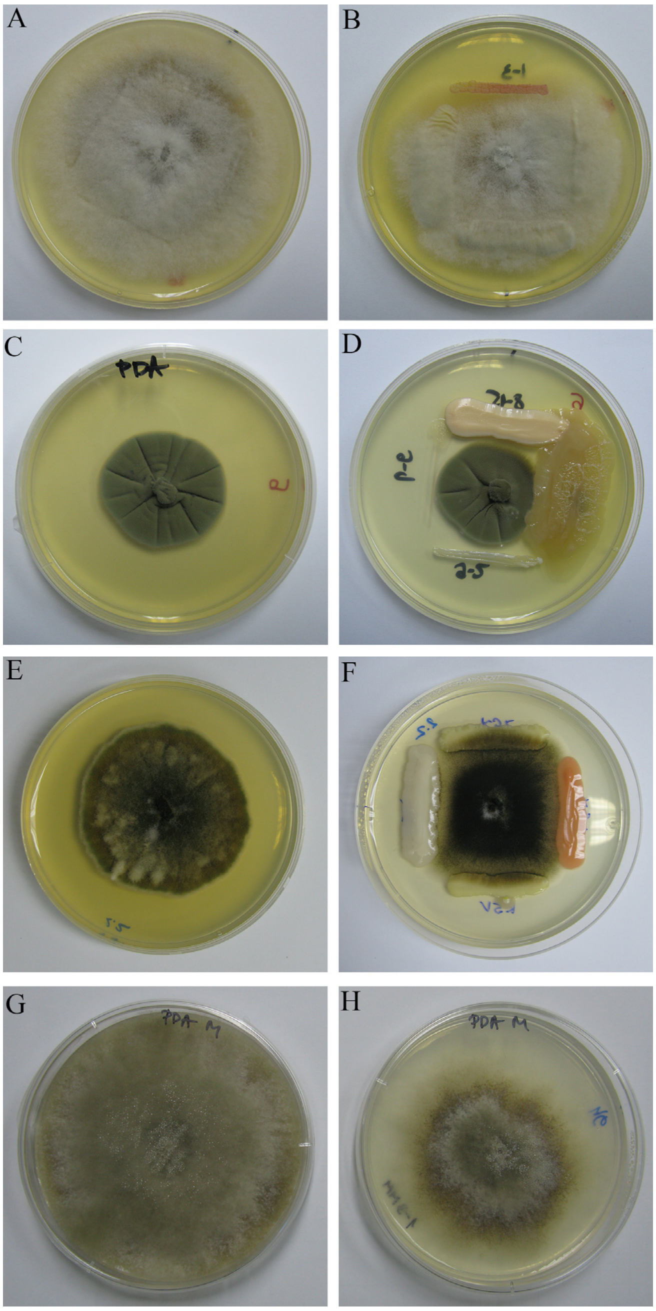

Figure S1: Dual culture method for determining the antagonistic potential of bacterial and yeast isolates. The activity of selected isolates against: *Botrytis cinerea* (A – control, B – treatment). *Cladosporium cladosporioides* (C – control, D – treatment), *Alternaria tenuissima* (E – control, F– treatment), and activity of *Hannaella luteola* V1/3 supernatant against *B. cinerea* (G – control, H– treatment) were shown.
